# IL-2RG as a possible immunotherapeutic target in CRC predicting poor prognosis and regulated by miR-7-5p and miR-26b-5p

**DOI:** 10.1186/s12967-024-05251-2

**Published:** 2024-05-08

**Authors:** Ehsan Gharib, Leili Rejali, Moein Piroozkhah, Elham Zonoobi, Parinaz Nasri Nasrabadi, Zahra Arabsorkhi, Kaveh Baghdar, Elahe Shams, Amir Sadeghi, Peter J. K. Kuppen, Zahra Salehi, Ehsan Nazemalhosseini-Mojarad

**Affiliations:** 1 Basic and Molecular Epidemiology of Gastrointestinal Disorders Research Center, Research Institute for Gastroenterology and Liver Diseases, Shahid Beheshti University of Medical Sciences, Tehran, Iran; 2 Department of Surgery, Leiden University Medical Center, Leiden, Netherlands; 3 Gastroenterology and Liver Diseases Research Centre, Research Institute for Gastroenterology and Liver Diseases, Shahid Beheshti University of Medical Sciences, Yeman Street, Chamran Expressway, P.O. Box: 19857-17411, Tehran, Iran; 4 Hematology, Oncology and Stem Cell Transplantation Research Center, Tehran University of Medical Sciences, Tehran, Iran; 5 Research Institute for Oncology, Hematology and Cell Therapy, Tehran University of Medical Sciences, Tehran, Iran

**Keywords:** Colorectal cancer, Interleukin-2 receptor gamma, Tumor-infiltrating lymphocytes, Hsa-miR-7-5p, Hsa-miR-26b-5p, Biomarker

## Abstract

Despite advances in treatment strategies, colorectal cancer (CRC) continues to cause significant morbidity and mortality, with mounting evidence a close link between immune system dysfunctions issued. Interleukin-2 receptor gamma (IL-2RG) plays a pivotal role as a common subunit receptor in the IL-2 family cytokines and activates the JAK-STAT pathway. This study delves into the role of Interleukin-2 receptor gamma (IL-2RG) within the tumor microenvironment and investigates potential microRNAs (miRNAs) that directly inhibit IL-2RG, aiming to discern their impact on CRC clinical outcomes. Bioinformatics analysis revealed a significant upregulation of IL-2RG mRNA in TCGA-COAD samples and showed strong correlations with the infiltration of various lymphocytes. Single-cell analysis corroborated these findings, highlighting IL-2RG expression in critical immune cell subsets. To explore miRNA involvement in IL-2RG dysregulation, mRNA was isolated from the tumor tissues and lymphocytes of 258 CRC patients and 30 healthy controls, and IL-2RG was cloned into the pcDNA3.1/CT-GFP-TOPO vector. Human embryonic kidney cell lines (HEK-293T) were transfected with this construct. Our research involved a comprehensive analysis of miRPathDB, miRWalk, and Targetscan databases to identify the miRNAs associated with the 3′ UTR of human IL-2RG. The human microRNA (miRNA) molecules, hsa-miR-7-5p and hsa-miR-26b-5p, have been identified as potent suppressors of IL-2RG expression in CRC patients. Specifically, the downregulation of hsa-miR-7-5p and hsa-miR-26b-5p has been shown to result in the upregulation of IL-2RG mRNA expression in these patients. Prognostic evaluation of IL-2RG, hsa-miR-7-5p, and hsa-miR-26b-5p, using TCGA-COAD data and patient samples, established that higher IL-2RG expression and lower expression of both miRNAs were associated with poorer outcomes. Additionally, this study identified several long non-coding RNAs (LncRNAs), such as ZFAS1, SOX21-AS1, SNHG11, SNHG16, SNHG1, DLX6-AS1, GAS5, SNHG6, and MALAT1, which may act as competing endogenous RNA molecules for IL2RG by sequestering shared hsa-miR-7-5p and hsa-miR-26b-5p. In summary, this investigation underscores the potential utility of IL-2RG, hsa-miR-7-5p, and hsa-miR-26b-5p as serum and tissue biomarkers for predicting CRC patient prognosis while also offering promise as targets for immunotherapy in CRC management.

**Graphical Abstract:**

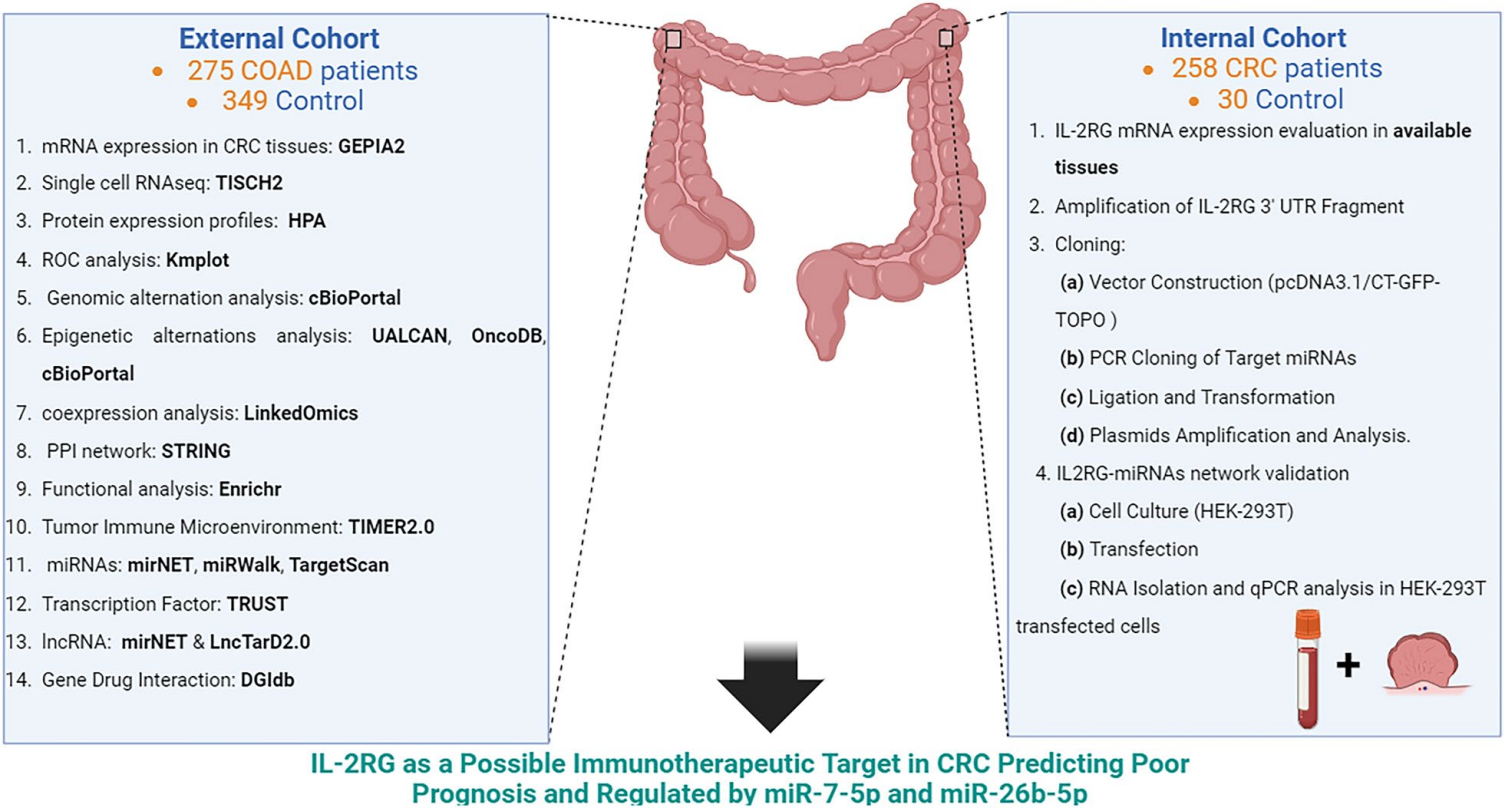

**Supplementary Information:**

The online version contains supplementary material available at 10.1186/s12967-024-05251-2.

## Introduction

Within the scope of cancer investigations, significant advances over the last century have illuminated the intricate interplay between the immune system and malignant cells [1]. These discoveries have served as the foundation for the concept of ‘immunosurveillance,’ representing the immune system’s capacity to identify and eliminate abnormal cells. Conversely, ‘immunoediting’ involves the reciprocal interaction and shaping of both the immune system and cancer cells, eventually leading to the growth and advancement of cancer [2]. Notably, resistance to immune attacks and the presence of pro-tumoral inflammation stand out as two pivotal hallmarks in cancer [3]. Highlighting its utmost importance, the immune contexture, which refers to the immune landscape at the cancer presentation site, has the potential to determine the prognosis for patients with colorectal cancer (CRC) [4, 5].

Cytokines, soluble proteins facilitating cell-to-cell communication, are pivotal in the dynamic interactions between immune and non-immune cells within the tumor microenvironment (TME) [6]. In the context of cancer, cytokines are central to shaping the migration patterns of immune cells into the tumor, ultimately influencing the immune profile within the TME [7]. Among cytokines, several interleukins (ILs) assume particular relevance in cancer development and progression. The multitude of cellular sources, receptors, signaling pathways, and dose dependency collectively define the pleiotropic role of ILs in cancer [6]. Importantly, ILs can exhibit cell-specific effects, influencing cancer initiation, tumor progression, and control. ILs can be categorized into various families with over 40 subfamily members [8]. ILs have attracted significant attention in recent years due to their unique roles in different cancer types. Of particular interest is their potential for innovative treatment strategies, especially in the treatment of CRC [9, 10].

The proliferation, maturation, and regulation of innate and adaptive immune cells are subject to strict control by IL-2 family cytokines. These cytokines, namely IL-2, IL-4, IL-7, IL-9, IL-15, and IL-21, utilize heteromeric receptors and share a common gamma chain (γC) receptor subunit, also known as the IL-2RG receptor [11]. The activation of immune cells by ILs depends on binding these cytokines to high-affinity receptors located on cell surfaces.

However, it is essential to note that activated ILs cannot exert their function until their respective cell surface receptors recognize them [12]. Each IL possesses a distinct receptor or set of receptors with which it can interact. This specificity is crucial as it allows for precise signaling within the immune system. The interaction between the IL and its receptor triggers a cascade of intracellular signaling events, commonly referred to as signal transduction [13].

Typically, the common receptor of the IL-2 family, known as IL-2RG, engages in the formation of heterodimers or heterotrimers with various other subunits, including IL-2Rα/β, IL-15Rα, IL-21Rα, IL-4Rα, IL-7Rα, and IL-9Rα [14]. Moreover, IL-2RG is crucial in transmitting signals via the Janus kinase (JAK)-signal transducer and activator of transcription (STAT) pathways [15, 16]. The IL-2RG receptor is encoded by the IL-2RG gene, situated on the X-chromosome q13.1. It is worth noting that based on research on the selective effect on the genetic landscape of tumor cells, different immunological contexts translate into differences in immunogenicity and that the selective effect of the immune system on cancer cells seems to be less based on the selection of specific mutational signatures and more on the selection of specific mutated genes with lower immunogenic potential [17], furthermore mutation in IL-2RG give rise to X-linked severe combined immunodeficiency disease (X-SCID), a condition marked by reduced or absent T cells, NK cells, and non-functional B cells [18, 19].

Although the IL-2RG gene has been relatively understudied in cancer research, emerging evidence highlights its role in tumorigenesis. IL-2RG influences malignant cell differentiation, activation, and proliferation, shaping the TME [20]. Particularly, IL-2RG expression has been identified in human gastric carcinomas and is associated with an unfavorable prognosis [21, 22]. In recent years, the study of miRNAs in cancer research has gained significant attention due to their involvement in various biological processes, including carcinogenesis [23]. Notably, various studies have identified that the expression levels of let-7a, miR-7-5p, miR-26b-5p, miR-128, miR-421, and miR-873 were significantly reduced in CRC [24–29].

Unraveling the intricate functions of IL-2RG receptors in the context of CRC is of paramount significance. It lays the foundation for developing targeted therapies that can augment the immune system’s efficacy in combating cancer and provide valuable insights for predicting the survival of CRC patients. To address this crucial objective, our study aims to illuminate the multifaceted roles of IL-2RG in CRC by leveraging high-throughput data analysis supported by rigorous experimental validation. Additionally, we delve into exploring and validating microRNAs (miRNAs) that target IL-2RG, adding a further layer of insight to our research.

## Results

### The mRNA expression landscape of IL-2RG in CRC tissues

**Fig. 1:**
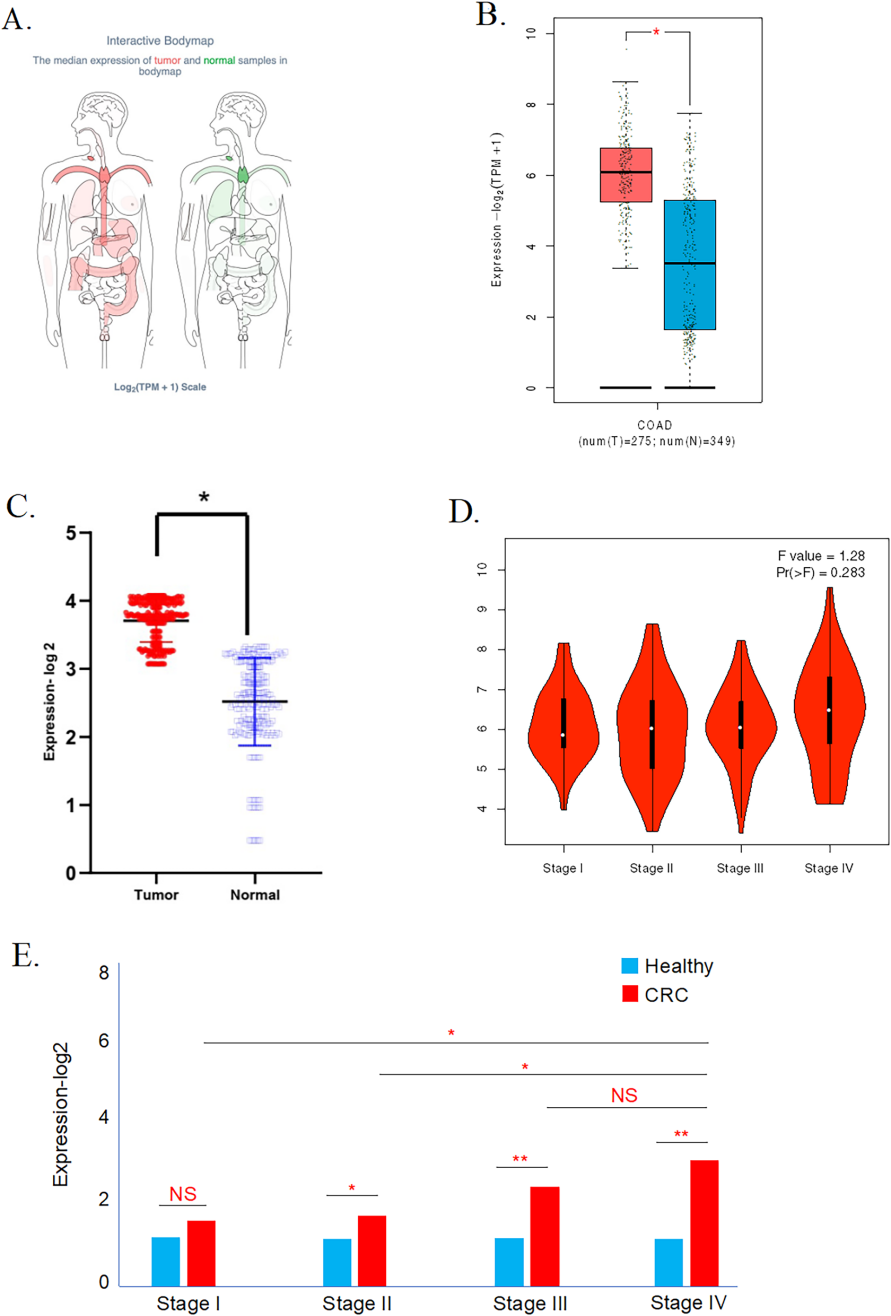
Expression analysis of the IL-2RG gene in CRC tissue. **A** Interactive body maps, **B**, **C** box plot of transcript expression of IL-2RG in TCGA-COAD (**B**), and data released from the internal cohort (**C**). **D**, **E** Correlations between the IL-2RG transcript expression with COAD TNM stage (**D**), and the independent CRC cohort pathological stage (**E**). *P < 0.05, **P < 0.01, ***P < 0.001. *P* < 0.05 was considered significant

In a rigorous external cohort designed for exploration of the IL-2RG transcriptomic landscape, we delineated distinctive patterns of IL-2RG mRNA expression across a spectrum of human malignancies versus their normal tissue counterparts. Leveraging the insights of the interactive BodyMap and Pancancer view, we discerned pronounced discrepancies in IL-2RG transcriptional expression, most saliently cancers within digestive system organs, encompassing the pancreas, stomach, and colon (Fig. 1A and Supplementary Fig. 1). Extending our scrutiny to a comparative transcriptomic assay, containing 275 COAD patients and 349 non-pathological controls drawn from the TCGA and GTEx repositories further underscored the conspicuous hyperexpression of the IL-2RG gene (Fig. 1B). Complementing these findings, our research cohort reflected a surge in IL-2RG mRNA levels in CRC patients as well (Fig. 1C).

To substantiate the observed enhancement of IL-2RG expression within COAD, we turned to the detailed immunohistochemical (IHC) staining available within the Human Protein Atlas (HPA) repository, comparing tumor tissues against non-neoplastic colonic tissues. The following analysis confirmed an upregulation of IL-2RG protein in COAD (Supplementary Fig. 2).

These findings support the idea that the IL-2RG gene shows different levels of expression in CRC compared to normal cells, highlighting its unique transcriptional patterns in this context.

### Correlations between the IL-2RG expression and clinicopathological features of CRC patients

The pathological stage of the tumor is a significant indication of the patient’s prognosis. In light of the external cohort, we undertook an incisive study of TCGA-COAD patients to decipher potential correlations between IL-2RG expression and the pathological stages of COAD. Our analysis spotlighted a trend wherein IL-2RG gene expression augmented concomitant with more advanced tumor stages, though the association was not statistically pronounced (p-value > 0.05, Fig. 1D).

This trajectory was mirrored in our research cohort, wherein expression of IL-2RG exhibited a statistically significant escalation in stage II and even more pronouncedly in stage III compared with healthy controls. Although stage IV patient data did not indicate a discernible divergence from stage III, it markedly exceeded the expression levels observed in stage I and II CRC specimens (Fig. 1E).

Further, the association between the expression of IL-2RG and various clinicopathological aspects of CRC patients was assessed. The result indicated that the IL-2RG was significantly correlated with the patient’s gender, location of the tumor, and pathological differentiation (all p-values < 0.05, Supplementary Table S1).

In aggregation, these insights underscore a tangible nexus between tissue IL-2RG expression and CRC pathological stages, suggesting its potential utility as a precise indicator for clinical staging guidance.

### Prognostic and diagnostic evaluation of IL-2RG expression in CRC patients

**Fig. 2:**
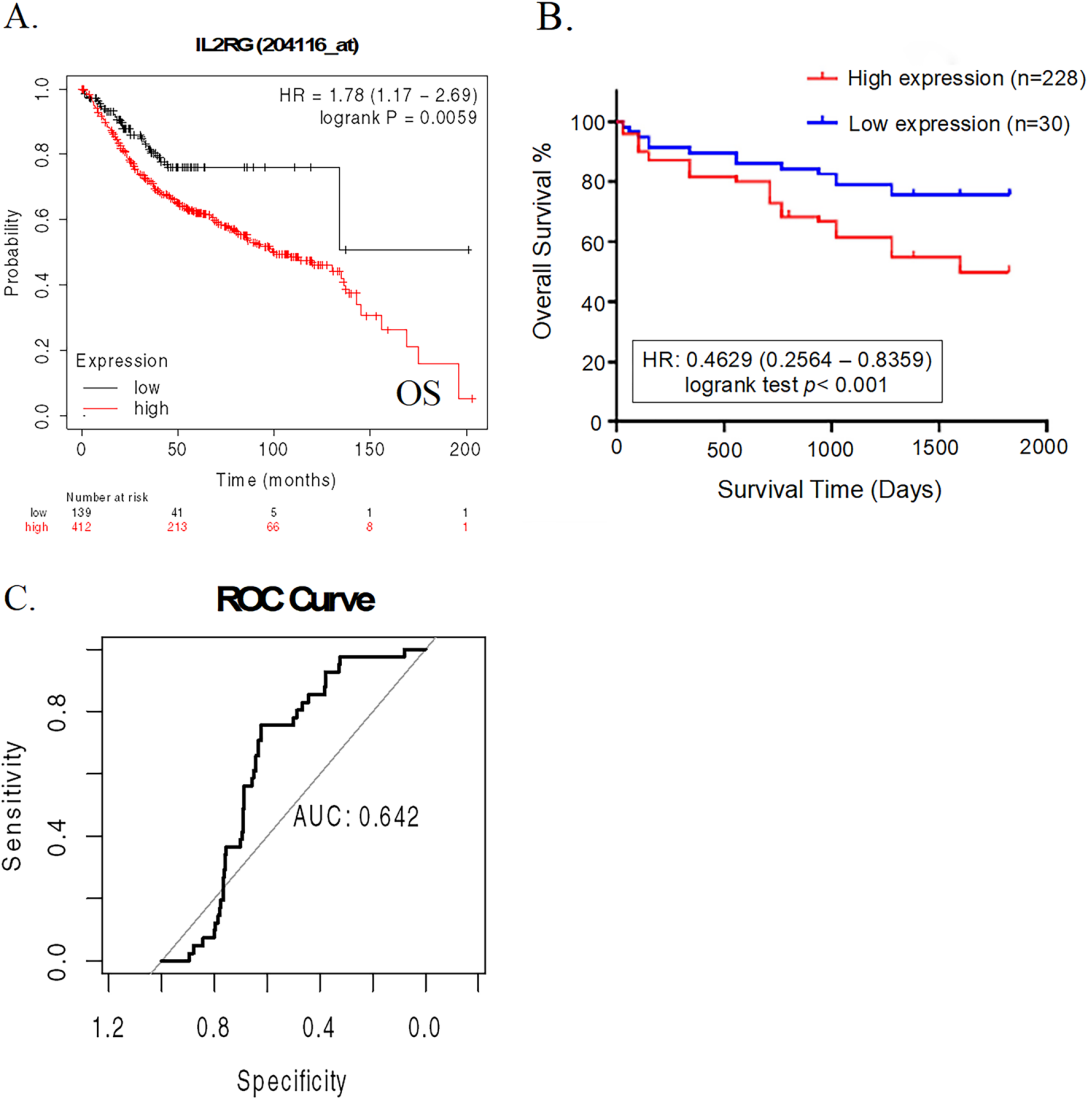
Prognostic and diagnostic evaluation of IL-2RG expression in CRC. **A** Association between IL-2RG transcript with overall survival of TCGA-COAD and **B** data released from the present cohort. **C** The diagnostic value of the IL-2RG transcript between COAD tissue and corresponding normal tissues

In our quest to discern the prognostic significance of IL-2RG within CRC, we meticulously investigated its expression concerning the clinical outcomes of patients, employing both the Kmplot database repository. As delineated in Fig. 2A, when patients were divided by mean expression of IL-2RG, the high IL-2RG expression group notably demonstrated a reverse correlation with the overall survival (OS) rate in the 551 COAD subjects included in the external cohort. Moreover, the IL-2RG expression was shown to have a significant reverse association with the OS rate in the 258 CRC subjects from our research cohort (Fig. 2B).

Transitioning to its diagnostic utility, a thorough examination of the TCGA dataset revealed that, as captured in the ROC curve, the diagnostic insight of IL-2RG expression levels, even though modest, remained distinguishable (AUC = 0.642, Fig. 2C).

### Comprehensive examination of genetic alterations within the IL-2RG

**Fig. 3:**
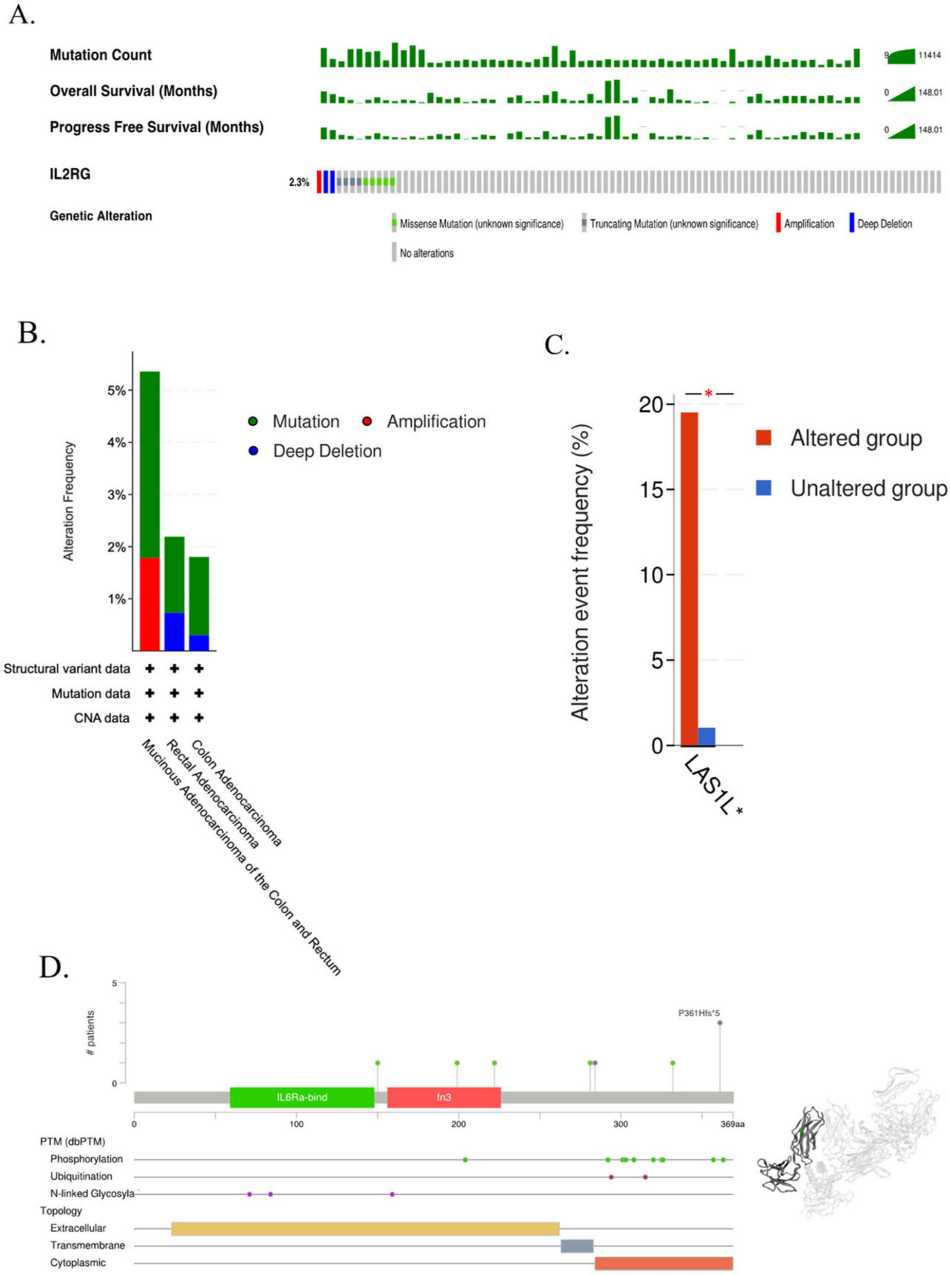
Analysis of IL-2RG genetic alteration in COAD. **A**, **B** The total alterations in the IL-2RG genes were assessed using a genome-wide COAD analysis in the cBioPortal database (TCGA, Firehose Legacy). **C** Co-occurrence mutation in IL-2RG altered patients compared to the unaltered group. **D** Mutation diagram and Post-translational Modification data of IL-2RG COAD across protein domains. *P < 0.05, **P < 0.01, ***P < 0.001. *P* < 0.05 was considered significant

Genomic aberrations play a cardinal role in oncogenesis, with altered genes often manifesting as pivotal prognostic biomarkers [30]. Undertaking to illuminate the genetic alteration landscape of IL-2RG in COAD as the external cohort designed, our initial exploration involved conducting a thorough analysis of the TCGA-PanCancer atlas using the cBioPortal Database. The results elucidated alterations in IL-2RG in 2.3% (equivalent to 12 out of 512) COAD patients (Fig. 3A, B). Proceeding further, we delved into co-mutation patterns within the cohort displaying IL-2RG alterations (41 cases) compared to the unaltered cohort (483 cases). This analysis highlighted a pronounced co-occurrence of the LAS1L gene alteration predominantly within the IL-2RG-altered cohort (Fig. 3C).

Aiming to chart the mutational signatures of IL-2RG across its constituent protein domains, we undertook an in-depth analysis, which revealed a septet of seven mutation sites (Fig. 3D).

In concordance with the Post-translational Modification (PTM) dataset delineated in Fig. 3D, phosphorylation emerged as the predominant PTM category. Remarkably, among the ten identified sites, the cytoplasmic region of IL-2RG was a recurrent locus for these modifications (Fig. 3D).

### Epigenetic regulation of IL-2RG expression through promotor methylation in COAD

**Fig. 4:**
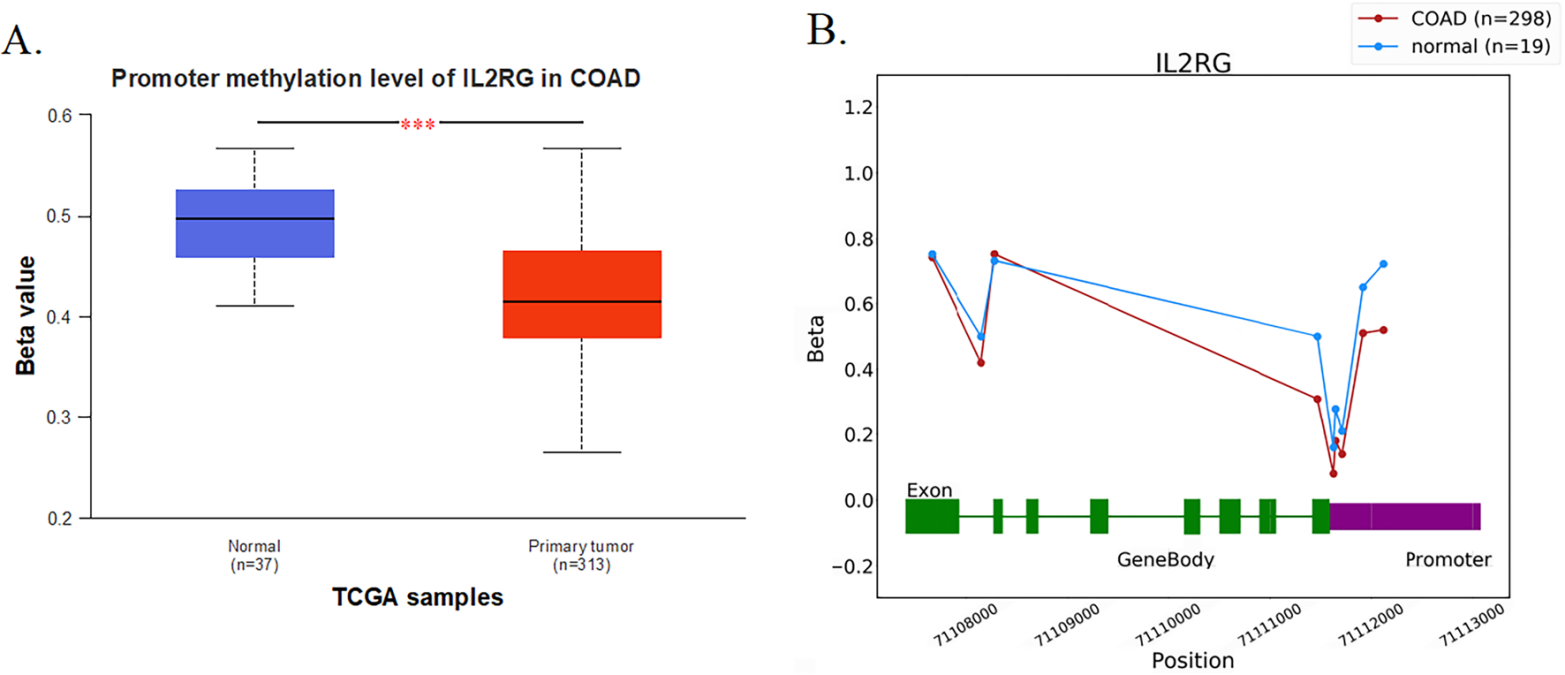
Analysis of the IL-2RG promotor methylation in COAD. **A**, **B** The methylation profile of the IL-2RG promotor in a cohort consisting of 313 COAD and 37 corresponding normal tissues based on the UALCAN database and 298 COAD and 19 corresponding normal tissues based on the ONCODB database have shown hypomethylation of this gene in COAD tissues. *P < 0.05, **P < 0.01, ***P < 0.001. *P* < 0.05 was considered significant

Epigenetic modifications, particularly DNA methylation, are pivotal in regulating gene expression. Aberrant methylation patterns are frequently implicated in oncogenesis, underscoring their potential significance in the cancer biology [31]. With this context, we endeavored to delineate the methylation profile of the IL-2RG promotor in a cohort consisting of 313 COAD and 37 corresponding normal tissues. Strikingly, the promotor region of IL-2RG manifested a marked hypomethylation in COAD samples as juxtaposed to their normal counterparts (Fig. 4A, B, and Supplementary Table 2). This observation emphasizes the prominent epigenetic control of IL-2RG expression via methylation. Further heightening our insight, a rigorous analysis revealed a robust inverse correlation between the expression and methylation of IL-2RG, unequivocally underscoring the repressive influence of methylation on the gene expression (Spearman correlation = − 0.54, p-value = 1.00e−40, Supplementary Fig. 3).

### Functional analysis of IL-2RG‐related pathways in COAD

**Fig. 5:**
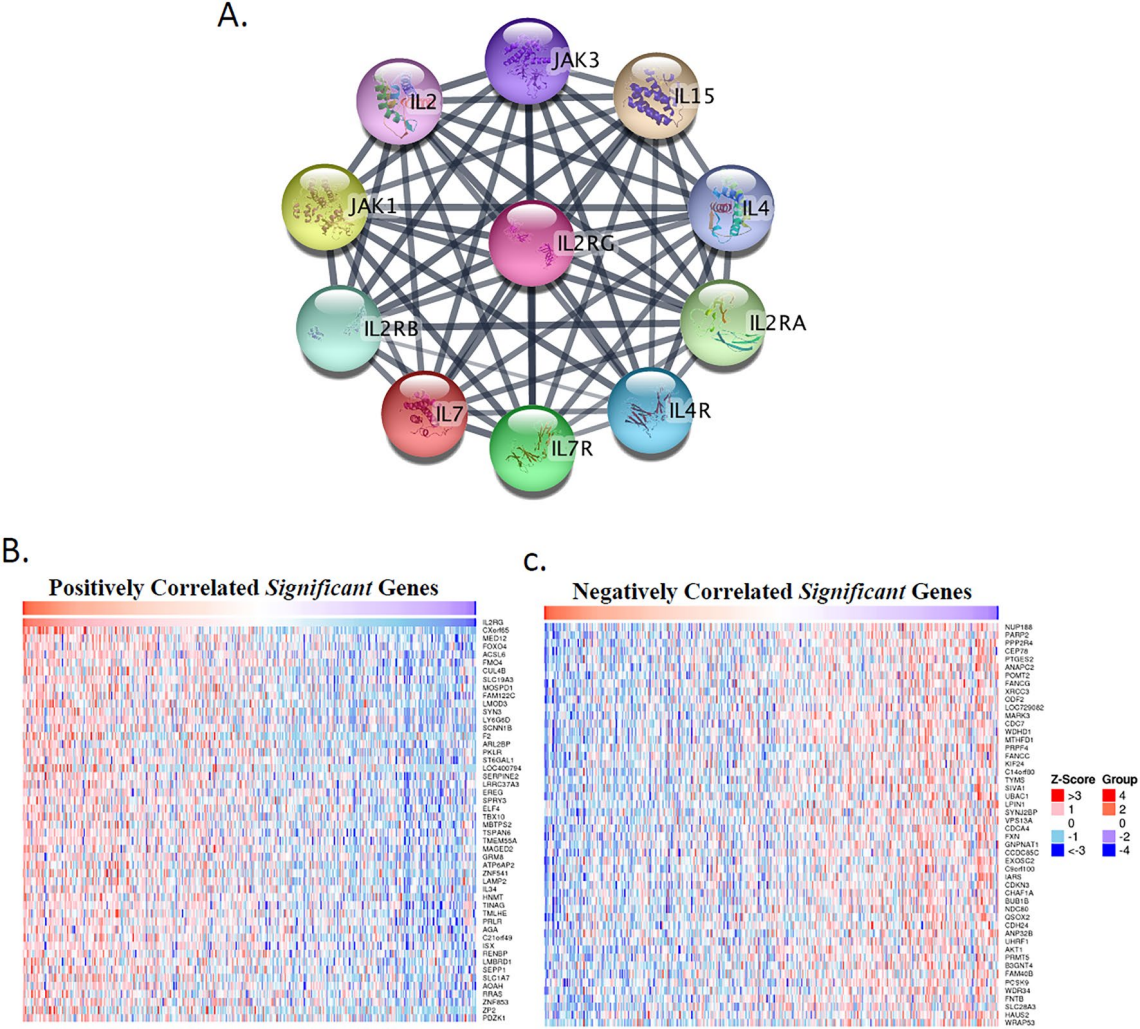
Protein–protein interaction (PPI) network, co-expression genes, and functional analysis of IL-2RG. **A** Predicting the PPI network of IL-2RG using the STRING database. **B**, **C** Heatmap demonstrating the positively (**B**) and negatively (**C**) correlated genes in COAD

To understand the functional roles of IL-2RG, a comprehensive analysis was conducted as the external cohort to identify interacting proteins and the most relevant genes of IL-2RG. To accomplish this, the experimentally validated Protein–Protein Interaction (PPI) network of IL-2RG was visualized using the STRING databases (Fig. 5A).

Besides, the top 100 co-related genes (50 positively and 50 negatively co-related genes) of IL-2RG in COAD were displayed by the heatmaps and volcano plots (Fig. 5B, C, and Supplementary Fig. 4 and Table S3). The strongest correlation of IL-2RG in COAD was noticed with four genes, including CXorf65, MED12, ACSL6, and FOXO4, respectively.

To uncover the potential biological characteristics of IL-2RG, enrichment analyses were conducted. The Gene Ontology (GO) enrichment analysis delineated a multitude of immunological functions associated with IL-2RG, prominently including mechanisms such as T-cell activation, negative regulation of leukocyte degranulation, neutrophil extravasation, CD4 receptor binding, and T-cell receptor complex. Further assessment through KEGG analysis revealed a significant enrichment in pathways indicative of immune cell signatures. Notably, pathways such as Natural killer cell-mediated cytotoxicity, T-cell receptor signaling pathway, Th1 and Th2 cell differentiation, and the chemokine signaling pathway have been identified (Supplementary Fig. 5). These results provide evidence of the role of IL-2RG in regulating lymphocytes and immune responses that are essential in tumorigenic processes.

### Correlation between the IL-2RG and the tumor immune microenvironment

**Fig. 6:**
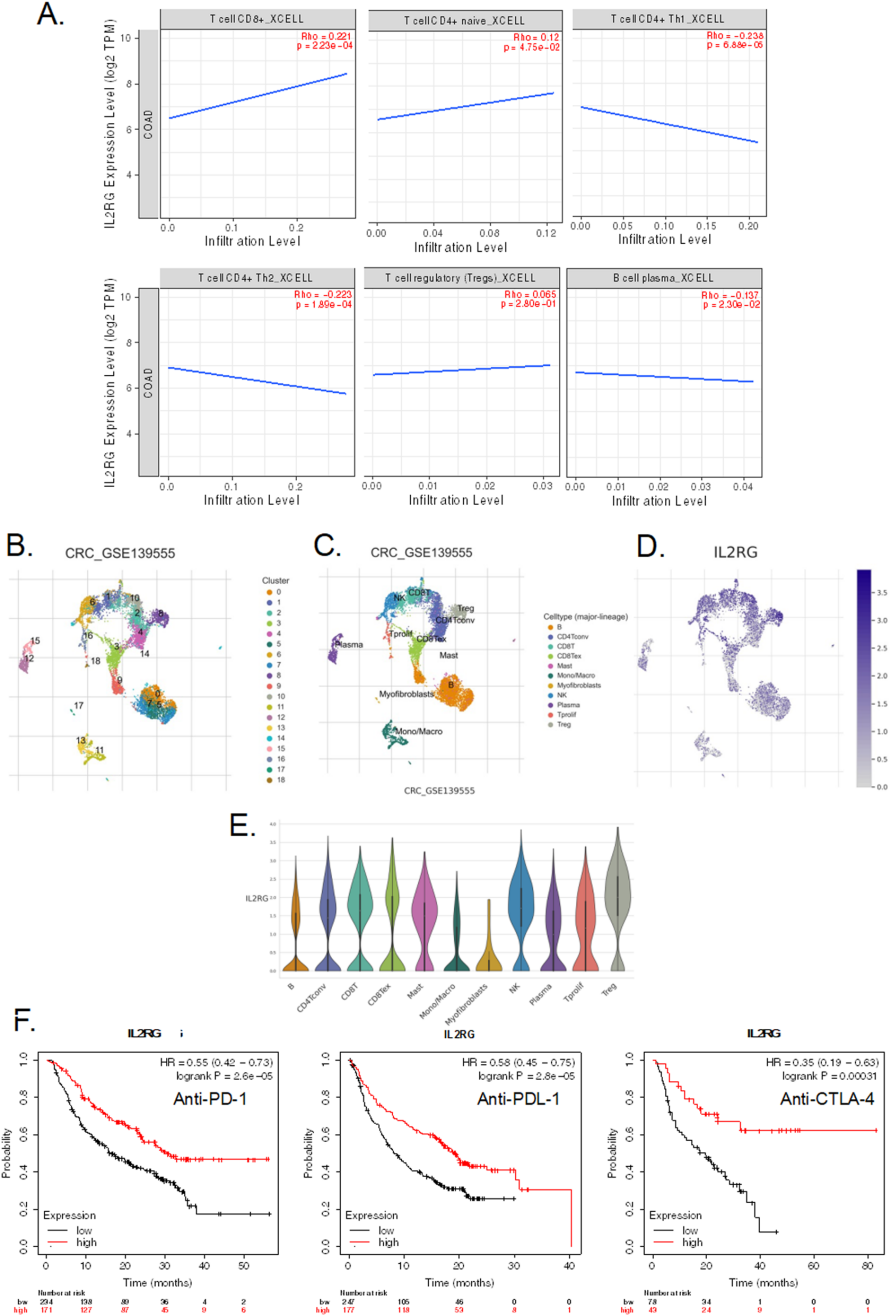
Correlation between the IL-2RG and the tumor immune microenvironment (TIME). **A** Association between the IL-2RG expression and various types of lymphocytes (CD8^+^ T-cells, CD4^+^ Naïve T-cells, CD4^+^ Th1 T-cells, CD4^+^ Th2 T-cells, and B-plasma cells) infiltration in COAD. **B**, **C** Classification and statistics of cell types in the CRC_GSE139555 single-cell sequencing data. **D** Distribution and expression of the IL-2RG in lymphocyte clusters. **E** Violin plots showing the IL-2RG expression levels across various TIME cells. **F** Kaplan–Meier plots predict patients’ overall survival in various cancers based on IL-2RG expression in different immunotherapy regimens (anti-PD-1, anti-PDL-1, and anti-CTLA-4). *P < 0.05, **P < 0.01, ***P < 0.001. *P* < 0.05 was considered significant

Given IL-2RG’s pivotal role in regulating lymphocytes, our study set out to evaluate its significance within the context of tumor-infiltrating lymphocytes. Initially, we investigated the correlation between IL-2RG gene expression and the infiltration of distinct lymphocyte subtypes in COAD patients. Our findings unveiled a positive correlation between IL-2RG expression and CD8^+^ T-cells (rho = 0.221, p-value = 2.23e−04) and CD4^+^ Naïve T-cells (rho = 0.12, p-value = 4.75e−02). Conversely, a negative correlation emerged with CD4^+^ Th1 T-cells (rho = − 0.238, p-value = 6.88e−05), CD4^+^ Th2 T-cells (rho = − 0.223, p-value = 1.89e−04), and B-plasma cells (rho = − 0.137, p-value = 2.30e−02) (Fig. 6A).

We also investigated the association of the IL2RG gene with immune cells via the TISIDB database. Based on the database, the IL2RG was associated with Act CD4 (rho = − 0.148, p-value = 0.00151), Th2 (rho = − 0.184, p-value = 7.73e−05), Mem B (rho = 0.134, p-value = 0.00407), CD56bright (rho = 0.118, p-value = 0.0115), CD56dim (rho = − 0.105, p-value = 0.0247), and monocyte (rho = 0.11, p-value = 0.0187); while the other associations were not significantly meaningful.

Subsequently, our investigation into the cellular localization of IL-2RG expression in CRC was based on the analysis of single-cell RNA-sequencing data from the CRC_GSE139555 dataset. Within this dataset, we identified a total of 19 distinct cell clusters representing eleven unique cell types (Fig. 6B, C). Notably, IL-2RG expression was predominantly observed in CD8+ T-cells, CD8+ exhausted T-cells, CD4+ conventional T-cells, Treg cells, and Natural killer cells, as opposed to other cell types within the TME (Fig. 6D, E).

Finally, our study delved into the impact of IL-2RG expression levels on the response to immunotherapy. We uncovered a positive association between IL-2RG expression and the prognosis of cancer patients undergoing various immunotherapy treatments, including those involving anti-PD1, anti-PD-L1, and anti-CTLA-4 (Fig. 6F). These collective results imply that IL-2RG might serve as a promising predictive biomarker for patients’ response to immunotherapy in diverse cancer types. Nevertheless, further evaluation of the prognostic value of IL-2RG expression in COAD patients undergoing immunotherapy is warranted.

### Identification of miRNAs targeting the IL-2RG

In cancer, microRNAs (miRNAs) are involved in the regulation of a variety of biological processes, such as cell cycle, differentiation, proliferation, apoptosis, stress tolerance, energy metabolism, and immune response [32]. miRNAs work as small guide molecules in RNA silencing, negatively regulating the expression of several genes both at mRNA and protein level, by degrading or inhibiting the translation of their target mRNAs [33]. The most studied function of miRNAs is the regulation of gene expression via binding to the 3′-untranslated regions (UTRs) of target mRNAs and inhibition of their translation [34]. To verify the interaction between IL-2RG mRNA and miRNAs, an internal cohort was designed, we excised and amplified the 3′ UTR of IL-2RG mRNA sequence from lymphocytes of CRC patients. Excision and sub-cloning accuracy were verified by PCR and sequencing analysis, (Supplementary Table 4), and an amplified 347bp PCR product was achieved from the total RNA (335bp of 3′ UTR of IL-2RG mRNA sequence + forward and reverse primers). In addition, the supplied primers, with a region covering the T7 promoter plus insert and upstream of the GFP gene (801 bp length), were used to identify and compare to the empty vector (463 bp length). The construction of the pcDNA3.1/CT-GFP-TOPO vector before and after ligation is illustrated in Supplementary Fig. 6.

Principally, our selection of miRNAs with the potential to interact with IL-2RG was initiated based on the literature review, we looked for miRNAs that downregulated in CRC compared to normal tissues and candidate six miRNA including let-7a, miR-7-5p, miR-26b-5p, miR-128, miR-421, and miR-873 [24, 27–29, 35]. Then, the interaction of these six candidate miRNAs with IL-2RG was further evaluated through various databases, including the miRPathDB [36], miRWalk [37], and Targetscan [38]. This analysis identified these six miRNAs exhibited complementary binding sites on the 3′UTR of IL-2RG mRNA (Supplementary Fig. 7).

**Fig. 7:**
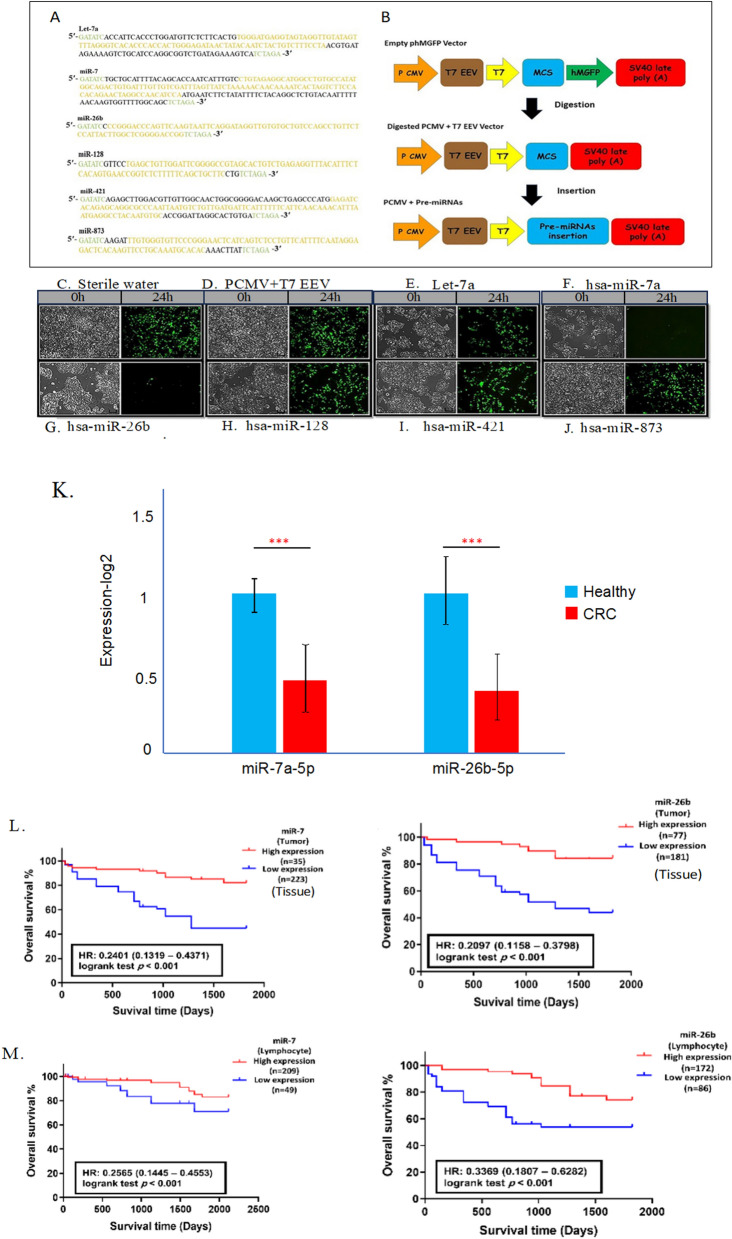
Cloning of the coding sequences of intended miRNAs into the phMGFP vector. **A** PCR amplified segment, including the coding sequence of pre-miRNAs along with the restriction digestion sites for EcorV (forward) and XbaI (reverse). The green parts show the restriction sites and the yellow parts indicate the coding sequences of target pre-miRNAs; **B** construction of the phMGFP vector before and after removing the hMGFP coding segment (PCMV + T7 EEV), and after pre-miRNAs insertion (PCMV + Pre-miRNAs). Transfection of the recombinant HEK-293T cells with the PCMV + Pre-miRNAs vectors; **C** HEK-293T cells transfected with sterile water and GFP level examined at start and after 24 h, transfected cells produced a strong GFP signal; **D** HEK-293T cells transfected with the PCMV + T7 EEV vector and GFP level examined at 0 h and after 24 h, the transfected cells produced a strong GFP signal; **E** HEK-293T cells transfected cells with the PCMV + let-7a vector, transfected cells produced less GFP signal; **F** HEK-293T cells transfected cells with the PCMV + miR-7 vector, transfected cells does not produce GFP signal; **G** HEK-293T cells transfected cells with the PCMV + miR-26b vector GFP level examined at 0h and after 24h, transfected cells does not produce GFP signal; **H** HEK-293T cells transfected cells with the PCMV + miR-128 vector GFP level examined at 0h and after 24h, transfected cells produced strong GFP signal, **I** HEK-293T cells transfected cells with the PCMV + miR-421 vector GFP level examined at 0h and after 24h, transfected cells produced strong GFP signal and **J** HEK-293T cells transfected cells with the PCMV + miR-873 vector GFP level examined at 0h and after 24h, transfected cells produced GFP signal. **K** The transcription analysis of hsa-miR-7-5p and hsa-miR-26b-5p miRNAs in lymphocytes of CRC patients and healthy individuals. Each value of qPCR was evaluated by three separate experiments. **L**, **M** Kaplan–Meier plots predict patients’ overall survival in CRC tissues (**L**) and CRC lymphocytes (**M**). *P < 0.05, **P < 0.01, ***P < 0.001. *P* < 0.05 was considered significant. phMGFP vector: vector encoding the Monster Green fluorescent protein; GFP: green fluorescent protein; EcorV: *Escherichia coli* restriction enzyme v; XbaI: *Xanthomonas badrii* restriction enzyme I; PCMV: derived from pUC cytomegalovirus; T7 EEV: T7 eukaryotic expression vector; HEK293: Human embryonic kidney 293

Next, pri-miRNA sequences were inserted into the PCMV + T7 EEV vector and overexpressed in the bacterial host cell. The sequencing analysis of the inserted fragments along with the construction of the PCMV + T7 EEV vector before and after ligation (PCMV + Pre-miRNAs vector) is shown in Fig. 7A, B. HEK-293T cells were transiently transfected with the plasmid constructs pcDNA3.1/CT-GFP-TOPO and the phMGFP for expression of the 3′ UTR of IL-2RG mRNA and intended miRNAs, respectively. After 48 h, HEK-293T cells were examined for GFP level. Almost all of the transfected cells produced a strong GFP signal. From the GFP expression, we were re-assured that the inserted 3′ UTR fragment was also successfully transcribed. Further, the quantity of the 3′ UTR of IL-2RG mRNA and the intended miRNAs were evaluated in each group (Supplementary Fig. 8).

**Fig. 8:**
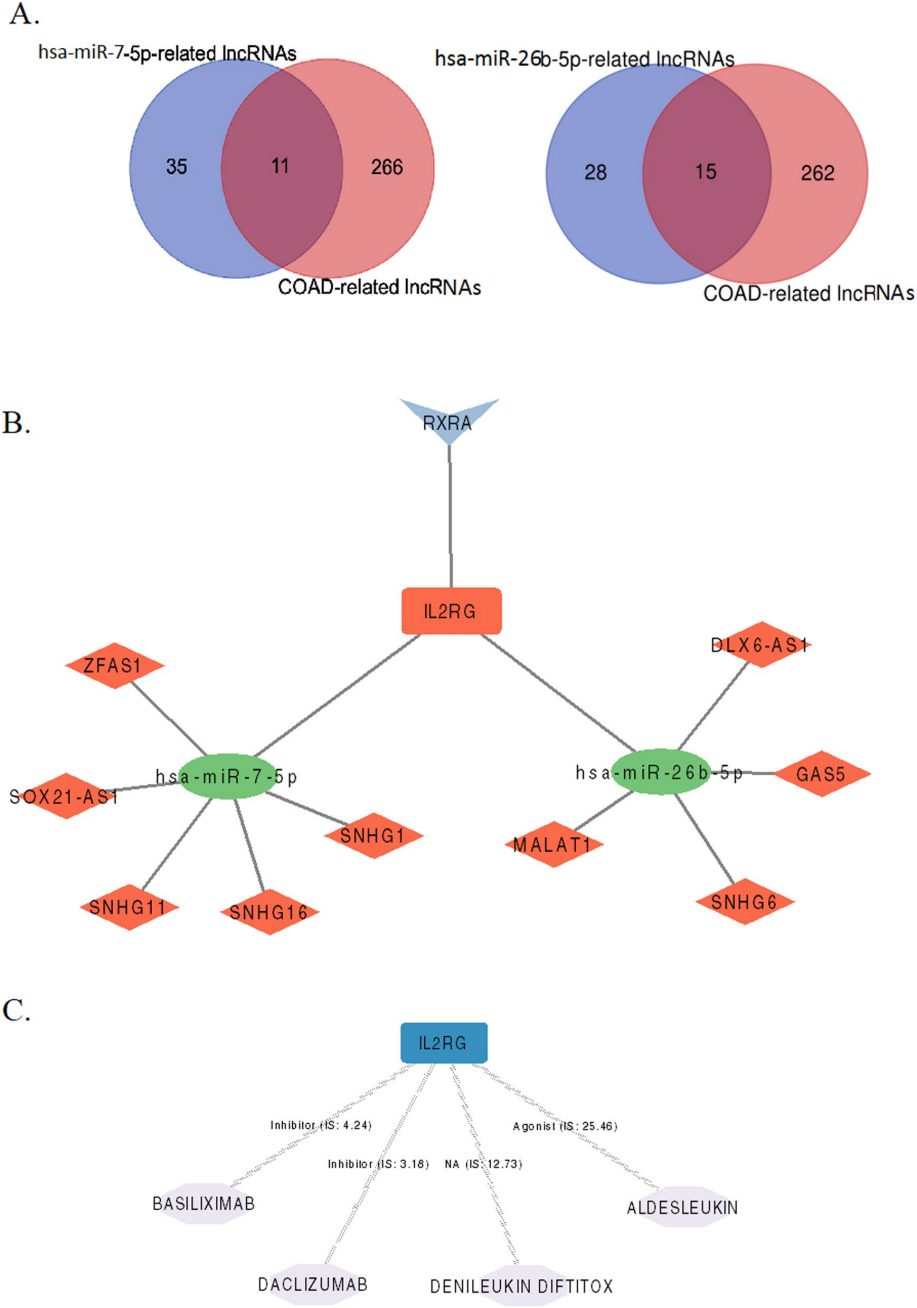
The regulatory and drug–gene network. **A** Intersection analysis of downstream lncRNAs associated with the two selected miRNAs (hsa-miR-7-5p and hsa-miR-26b-5p) and with the COAD-related lncRNAs. **B** Transcription factor—IL-2RG gene interaction and competing endogenous RNA network, **C** drug–gene interaction network. V shape: transcription factor, round rectangle: mRNA, ellipse: microRNA, diamond: long non-coding RNA, red shape: upregulation, green shape: downregulation, and hexagon: drug. *Networks were constructed using the Cytoscape version 3.10.1

To investigate the capacity of previously identified miRNAs to bind and inhibit IL-2RG mRNA, we categorized the transfected cells into distinct groups (Fig. 7C–J). Each subset was exposed to a unique miRNA-expressing plasmid. We postulated that successful binding between a miRNA and its complementary site on the 3′ UTR of IL-2RG mRNA would hinder the translation process, thereby inhibiting GFP production. A day post-transfection (24 h), a notable decline in GFP expression was observed in cells transfected with miR-7a-5p and miR-26b-5p (Fig. 7F, G respectively). Conversely, other groups—including those with let-7a (Fig. 7E), miR-128 (Fig. 7H), miR-421 (Fig. 8I), and miR-873 (Fig. 7J), as well as cells treated with the pCMV + T7 EEV plasmid, those receiving only sterile water (Fig. 7C), and the untreated cells (serving as the negative control)—showed no perturbation in GFP expression. Based on these findings, hsa-miR-7-5p and hsa-miR-26b-5p were selected for subsequent analyses.

### Expression profiling and clinicopathological correlation of hsa-mir-7-5p and hsa-mir-26b-5p in CRC patients

Initially, we quantified the expression profiles of hsa-miR-7-5p and hsa-miR-26b-5p in lymphocytes derived from our available eligible CRC patients and healthy controls. As delineated in Fig. 7K, both hsa-miR-7-5p and hsa-miR-26b-5p exhibited significant downregulation in lymphocytes isolated from CRC patients. Subsequently, we surveyed the association between the expression levels of miR-7-5p and miR-26b-5p with the clinicopathological characteristics of CRC patients. This was accomplished using qPCR analysis on samples from both tumor tissues and lymphocytes. Based on the median expression of the miRNAs under study, the cohort of 258 CRC patients was stratified as follows: miR-7-5p (low expression: n = 109; high expression: n = 49) and miR-26b-5p (low expression: n = 172; high expression: n = 86). Comprehensive analysis indicated that the expression patterns of miR-7-5p and miR-26b-5p in tumor tissues and patients’ lymphocytes were significantly associated with parameters such as tumor location, tumor differentiation, TNM staging, presence of metastasis, and IL-2RG status (all p-values < 0.05) (Supplementary Tables 5 and 6; respectively).

### Prognostic significance of miR-7-5p and miR-26b-5p expression in available colorectal patients

To elucidate the prognostic implications of hsa-miR-7-5p and hsa-miR-26b-5p expression of CRC patients, the Kaplan–Meier survival analysis was conducted. Notably, we found that elevated expression levels of these miRNAs in both tumor tissues and lymphocytes are significantly correlated with a more favorable OS in our CRC patient (Fig. 7L, M).

Univariate Cox regression analysis indicated that diminished levels of miR-7-5p (p-value < 0.001, HR = 0.614) and miR-26b-5p (p-value < 0.001, HR = 0.303) in Lymphocytes possess robust prognostic value in discerning CRC cases (Supplementary Table 7). On the other hand, multivariate Cox regression analysis demonstrated that reductions in miR-7-5p (p-value = 0.029, HR = 1.292, 95% CI 0.339–2.184) and miR-26b-5p (p-value = 0.041, HR = 0.586, 95% CI 0.255–3.314) expressions, independent of other assessed clinicopathological parameters, carry the most significant unfavorable prognostic values (Supplementary Table 7).

### IL-2RG regulatory network construction

In our quest to elucidate the regulatory dynamics of IL-2RG in the designed external cohort, a focused examination was conducted utilizing the TRRUST Database to identify potential TFs; notably unearthing RXRA as a pivotal activator of IL-2RG.

The rapidly growing field of cancer biology is increasingly spotlighting the competing endogenous RNA (ceRNA) as a groundbreaking regulatory mechanism, capturing the attention of researchers globally [39–42].

To augment our understanding of how lncRNAs contribute to the regulation of IL-2RG in the context of the ceRNA network involving hsa-miR-7-5p and hsa-miR-26b-5p, we aimed to create a detailed lncRNA-miRNA-mRNA (ceRNA) network. We retrieved the COAD-related lncRNAs from the LncTarD2.0 database (Supplementary Table 8). Subsequently, we collected the downstream lncRNAs associated with the two selected miRNAs (hsa-miR-7-5p and hsa-miR-26b-5p) and compared them with the COAD-related lncRNAs we obtained earlier (Supplementary Table 9). The overlapping 26 lncRNAs (eleven lncRNAs were associated with hsa-miR-7-5p, and 15 lncRNAs were associated with hsa-miR-26b-5p) were identified (Fig. 8A). Out of these 26 lncRNAs, we focused on identifying upregulated ones in COAD. To do this, we analyzed the expression levels of these 26 lncRNAs in the TCGA dataset. As a result, we discovered that nine lncRNAs (five lncRNAs for hsa-miR-7-5p and four lncRNAs for hsa-miR-26b-5p) were significantly upregulated in COAD tissues compared to normal tissues (Supplementary Fig. 9).

Further, we constructed and visualized the TF-gene and ceRNA networks using the Cytoscape version 3.10.1 tool (Fig. 8B).

At last, to investigate whether hsa-miR-7-5p and hsa-miR-26b-5p could regulate the co-expressed genes associated with IL-2RG, we leveraged the mirTarBase database to pinpoint mRNA targets of hsa-miR-7-5p and hsa-miR-26b-5p. Subsequently, we overlapped these targeted mRNAs with the 100 previously identified correlated genes using a Venn diagram. Our analysis revealed intriguing results: three mRNAs (EXOSC2, KIF24, and UHRF1) were found to be common between the correlated genes and miRNA-26b targets. Similarly, eight mRNAs (FANCC, CHAF1A, ELF4, TYMS, GRM8, ODF2, WDHD1, and AKT1) were shared between the correlated genes and miRNA-7 targets. This intersection sheds light on potential regulatory pathways implicating these miRNAs in IL-2RG-related processes. (Supplementary Fig. 10).

### Drug–gene interaction analysis

The investigation into potential drug–gene interactions leveraged the robust capabilities of the Drug–Gene Interaction Database (DGIdb). This analysis facilitated the identification of several drugs exhibiting interactions with IL-2RG, as detailed in Fig. 8C. This network showcases that the drugs BASILIXIMAB and DACLIZUMAB function as inhibitors, and ALDESLEUKIN serves as an agonist of IL-2RG, but the specific type of interaction involving DENILEUKIN has not been recorded (Supplementary Table 10).

## Discussion

Herein, through a comprehensive approach combining integrative bioinformatics analysis with rigorous experimental validation, we present a novel tumor immune-associated gene, defined as IL-2RG. Our study unveils the aberrant transcript and protein expression of IL-2RG, establishing a significant association with the prognostic outcomes of patients afflicted with COAD. Specifically, we reveal a positive correlation between IL-2RG expression and the infiltration of diverse lymphocyte subtypes in CRC. Furthermore, we analyze the relationship between IL-2RG expression and the predictive potential for various immunotherapy regimens. Additionally, we investigate miRNAs that directly modulate IL-2RG expression in the serum of CRC patients and assess their prognostic value. Notably, we undertake a pioneering exploration of genetic alterations, epigenetic regulation, regulatory network analysis, and the biological functions of IL-2RG within the context of CRC.

The IL-2RG gene encodes a protein known as the common γ chain (γC), a pivotal component of various immune system receptors. γC plays a fundamental role in developing and maintaining T cells and natural killer cells [43]. When defects in γC occur, resulting in X-SCID disease or JAK3 disruption, a downstream signaling molecule of IL-2RG, it can impair the development of B-cells. Even in cases where T-cell function is successfully restored through hematopoietic stem cell transplantation, B-cells with host-derived IL-2RG/JAK3 deficiencies often remain functionally impaired. This is primarily due to their inability to respond to the γC-dependent cytokine IL-21, which plays a critical role in the final stages of B-cell maturation and their differentiation into antibody-secreting plasmablasts [44].

Our current study uncovered a noteworthy pattern of IL-2RG overexpression, which displays an inverse relationship with patient survival, both within CRC tissues and lymphocytes. Furthermore, we noted a progressive increase in serum IL-2RG expression as TNM stages advanced.

Although research on IL-2RG in solid tumors remains limited, a few fundamental studies have hinted at the gene’s significance in oncogenesis. For example, Woods et al. [45] conducted experiments using a murine model in which stem cells from wild-type or mutant IL-2RG^−/−^ mice were transduced with significant amounts of human IL-2RG via a lentiviral vector. They made a significant observation that these transplanted mice exhibited a heightened incidence of tumors, positing that IL-2RG itself was contributing to tumorigenesis [45]. In another study, a transcript characterization of pancreatic intraepithelial neoplasia (PanINs) and normal pancreatic duct samples through RNA-seq revealed that IL-2RG overexpression played a role in the in vivo growth of pancreatic cancer cells [46]. A separate investigation disclosed the upregulation of IL-2RG in gastric cancer (GC) tissues, with high IL-2RG levels predicting a poorer OS. This suggests that IL-2RG may indeed have a role in developing human GC [21, 22]. These collective findings, consistent with our results, reinforce the significance of IL-2RG in cancer biology.

An intriguing inquiry arises regarding the potential proto-oncogenic role of IL-2RG in CRC. Proto-oncogenes are typically genes responsible for regulating essential cellular processes, including cell proliferation, differentiation, and various physiological activities. When these proto-oncogenes undergo abnormal activation, they can contribute to carcinogenesis. This activation can occur through a range of mechanisms, including genetic alterations such as gene translocations, amplifications, or point mutations, as well as epigenetic modifications like methylation [47, 48]. Cancer is fundamentally a genetic disorder that arises from the gradual accumulation of genetic aberrations affecting genes involved in multiple oncogenic pathways [49]. Our investigation unveiled that approximately eight percent of patients with COAD exhibited genetic alterations within the IL-2RG gene, predominantly characterized by missense mutations, amplifications, and deep deletions.

It is noteworthy that over 200 mutations have been cataloged in the IL-2RG gene thus far, with a preponderance of single-base substitutions (comprising missense and nonsense mutations), followed by splice site mutations, deletions, and insertions [50]. As genetic anomalies contribute to mRNA dysregulation, it is conceivable that these genetic alterations may prompt the overexpression of IL2RG mRNA [51, 52]. Moreover, our study unveils a compelling association between alterations in the LAS1L gene and the IL-2RG-altered cohort. LAS1L, a prominent member of the 5FMC (Five Friends of Methylated Chtop), plays a pivotal role in ribosome biogenesis and is intricately linked to TP53-mediated cell cycle control and autophagy [53, 54]. The emerging understanding of LAS1L’s significance in tumor progression is underscored by recent findings from the Samant et al. investigation, demonstrating that the knockdown of LAS1L results in reduced proliferation and diminished metastatic potential in triple-negative breast cancer cells [55].

Furthermore, our study demonstrated a heightened IL-2RG expression in COAD patients, which may be attributed to promoter hypomethylation relative to normal tissues. To ascertain this possibility, further validation through experiments, such as IL-2RG gene silencing or comprehensive methylation studies, is warranted.

Our present study provides compelling evidence regarding the immunological functions of IL-2RG in relation to COAD. Notably, IL-2RG exhibits positive associations with CD8^+^ T-cells and CD4^+^ Naïve T-cells while manifesting negative correlations with CD4^+^ Th1 T-cells, CD4^+^ Th2 T-cells, and B-plasma cells. These findings strongly suggest that IL-2RG likely plays a significant role in regulating immune processes pertinent to cancer, thus potentially influencing immunotherapeutic strategies. In a parallel study by Zhang et al. [56], it was demonstrated that elevated expression of IL-2RG, IL-2RA, IL-7R, and IFN-G may assume a pivotal role in driving melanoma metastasis, primarily through the augmentation of intratumoral regulatory T-cell proportions, mainly facilitated by the activation of the JAK-STAT signaling pathway [56].

Immunotherapies, such as CTLA-4 and PD-1/PD-L1 inhibitors, represent innovative and promising approaches to cancer treatment. They have shown great potential in gastrointestinal malignancies, particularly advanced diseases [57–59]. Nevertheless, a substantial challenge arises as only a minority of CRC patients experience a sustained clinical response to immunotherapy [60]. The presence of specific immune cells, particularly T-cells, within the TME, has been linked to an increased likelihood of enduring positive responses to immunotherapies [61]. Thus, we conducted further investigations to examine the connection between IL-2RG expression and the effectiveness of cancer immunotherapies. Our findings revealed that cancer patients with higher IL-2RG expression derive greater benefits from immunotherapies involving anti-PD1, anti-PD-L1, and anti-CTLA-4 treatments. Recent research endeavors have focused on assessing the response to anti-cancer therapy through the perspective of IL-2RG. For instance, in patients with pancreatic ductal adenocarcinoma (PDAC), Piper et al. revealed that the appropriate response to radiation therapy (RT) is characterized by increased levels of IL-2Rb and IL-2Rg, alongside diminished IL-2Ra expression [62].

Accumulating evidence underscores the functional significance of dysregulated miRNAs in driving cellular alterations, oncogenesis, and the overall survival of CRC patients [63]. In this study, we have identified and confirmed that hsa-miR-7-5p and hsa-miR-26b-5p directly target IL-2RG. Both hsa-miR-7-5p and has-miR-26b-5p are found to be downregulated in the serum of patients with CRC, and those with higher expressions of these miRNAs exhibit improved OS. Several studies have demonstrated that hsa-miR-7-5p acts as a suppressor in the development and progression of various cancers, including melanoma [64], non-small cell lung cancer [65], and breast cancer [66]. In the context of CRC, hsa-miR-7-5p emerges as a critical tumor suppressor, influencing processes such as cell proliferation, apoptosis, migration, invasion, and responsiveness to chemotherapy and radiotherapy [24]. Furthermore, one study has revealed that hsa-miR-26b-5p enhances the sensitivity of CRC cells to 5-FU in vitro and augments the efficacy of 5-FU in inhibiting tumor growth [26]. Another investigation has indicated that miR-26b restrains cell aggressiveness by regulating FUT4 in CRC [67].

Competing endogenous RNA (ceRNA) networks have emerged as pivotal players in the onset and progression of various cancer types [61]. To predict ceRNA networks specific to colorectal adenocarcinoma (COAD), our investigation has unveiled interactions between long non-coding RNAs (lncRNAs), including ZFAS1, SOX21-AS1, SNHG11, SNHG16, and SNHG1, with hsa-miR-7-5p. Similarly, we’ve identified interactions between lncRNAs DLX6-AS1, GAS5, SNHG6, and MALAT1 with hsa-miR-26b-5p in the context of COAD. We strongly recommend the validation of these networks within the CRC framework through experimental methodologies, such as dual-luciferase activity reporter assays.

This study comes with certain limitations that need to be acknowledged. To begin, the findings regarding the oncogenic and immunological functions of IL-2RG are derived from bioinformatics analyses of multiple online datasets, underscoring the need for further experimental validation to substantiate its involvement in cancer-related processes. This validation should encompass in vitro and in vivo experiments aimed at elucidating the impact of IL-2RG on the proliferation and migration of CRC patients. Additionally, investigating the influence of IL-2RG expression on the response to immunotherapy within a cohort of CRC patients presents an enticing avenue for future research.

In summary, our study has demonstrated that the serum levels of IL-2RG, hsa-miR-7-5p, and hsa-miR-26b-5p hold promise as non-invasive prognostic biomarkers for CRC patients. Furthermore, given the immunological role of the IL-2RG gene, we propose that it may serve as a valuable biomarker or a potential target for immunotherapy in CRC patients.

## Materials and methods

### Comparison of differential expression of IL-2RG between cancerous and normal tissues

The IL-2RG mRNA expression differences between COAD tissues and adjacent normal tissues were compared using the Gene Expression Profiling Interactive Analysis 2 database (GEPIA2, http://gepia2.cancer-pku.cn/#index) [68]. Furthermore, the differential IL-2RG protein expression profiles were explored through analysis of the Human Protein Atlas database (HPA, https://www.proteinatlas.org) [69].

### Survival analysis and receiver operating characteristic (ROC) analysis

The prognostic value of IL-2RG, including overall survival, was evaluated at a COAD level by plotting Kaplan–Meier (KM) curves using the Kaplan–Meier Plotter database (Kmplot, https://kmplot.com/analysis/) [70]. The log-rank p-value and hazard ratio (HR) were calculated to determine the prognostic value of IL-2RG. A p-value of smaller than 0.05 was considered to be of statistical significance.

The diagnostic potential of IL-2RG in COAD was assessed utilizing area under the ROC curves (AUC) based on TCGA datasets retrieved from the OncoDB database (https://oncodb.org) [71]. Specifically, AUC > 0.9 corresponds to an excellent level of prediction accuracy, while 0.7 > AUC > 0.6 is linked to a modest level of prediction accuracy.

### Analysis of the genomic alteration of IL-2RG in COAD

The COAD (TCGA, PanCancer project) analysis was carried out on the IL-2RG genomic alteration landscape, comprising mutation, amplification, and deep deletion, using the Cancer Types Summary module of the cBioPortal online web tool (https://www.cBioPortal.org/) [72]. The mutation landscape of IL-2RG protein and co-occurring mutations were also analyzed by this database.

### Investigation of the epigenetic modulation of IL-2RG in COAD

The promotor methylation of IL-2RG in COAD and normal tissues was analyzed in TCGA dataset using the OncoDB and the University of Alabama at Birmingham CANcer data analysis portal (UALCAN, https://ualcan.path.uab.edu) [73]. The significance of differences was determined using a student’s t-test, in which a P-value < 0.05 was considered significant.

### Functional analysis

The STRING platform (https://string-db.org) was utilized to develop the protein–protein interaction (PPI) network diagrams on the IL-2RG [74]. The study was followed by an investigation into identifying the positive and negative gene correlations with IL-2RG across the TCGA-COADREAD RNAseq data using the LinkedOmics database (https://www.linkedomics.org/login.php) [75]. Subsequently, a comprehensive identification of related gene ontology (GO) terms and pathways from the Kyoto Encyclopedia of Genes and Genomes (KEGG) was undertaken. In this process, a hundred IL-2RG-correlated genes were obtained from the ARCHS4 website (https://maayanlab.cloud/archs4/) and were further analyzed for enrichment using the Enrichr database (https://maayanlab.cloud/Enrichr/), facilitating a deeper exploration into the biological significance and pathway associations of the IL-2RG gene [76, 77]. Finally, the visual representation of the KEGG and GO terms, which include Biological Process (BP), Molecular Function (MF), and Cellular Component (CC), was created using the “ggplot2” package.

### Tumor immune microenvironment

To assess the relationship between IL-2RG expression and the infiltration of various lymphocyte subtypes, we initially leveraged the Tumor Immune Estimation Resource 2.0 database (TIMER2.0, http://timer.cistrome.org/). TIMER2.0 is a valuable tool that enables the comprehensive exploration of immune cell infiltration in tumor tissues, providing insights into the immunological context of cancer [78].

Subsequently, we employed the Tumor Immune Single-Cell Hub (TISCH2, http://tisch.comp-genomics.org/home/). This comprehensive online repository offers a platform for a systematic exploration of TME heterogeneity by single-cell sequencing analysis, drawing on diverse datasets and cell types [56]. In our current study, we delved into the cellular localization of IL2-RG expression using the CRC_GSE139555 dataset.

Lastly, we evaluated the prognosis of immunotherapy recipients across a range of cancer types, including Bladder cancer (n = 73), Esophageal adenocarcinoma (n = 103), Glioblastoma (n = 28), Hepatocellular carcinoma (n = 22), Head and Neck Squamous Cell Carcinoma (HNSCC, n = 5), Melanoma (n = 423), Non-Small Cell Lung Cancer (NSCLC, n = 21), Small Cell Lung Cancer (SCLC, n = 22), and Urothelial cancer (n = 348). This analysis was based on the expression levels of IL-2RG, categorized as low or high, and conducted using the Kmplot database (https://kmplot.com/analysis/) [70].

### Gene regulatory network

To identify transcription factors that regulate the IL-2RG gene, we used the Transcriptional Regulatory Relationships Unraveled by Sentence-based Text mining database (TRRUST, https://www.grnpedia.org/trrust/). Further, the Long non-coding RNAs (lncRNAs) related to identified miRNAs were harvested from the mirNET database (https://www.mirnet.ca/) [79]. The LncTarD2.0 database (https://lnctard.bio-database.com) was used to identify COAD-related lncRNAs and analyze the expression of lncRNAs in COAD compared to normal tissues [80]. The Cytoscape version 3.10.1 was used to construct and visualize networks.

### Clinical samples

Tumor tissue and blood specimens were collected from a total of 258 Iranian colorectal cancer (CRC) patients and 30 healthy individuals over the course of a 10-year period, from January 2012 to February 2022. These patients had been previously diagnosed and their cases were approved by the Research Institute for Gastroenterology and Liver Diseases (RIGLD) at Taleghani Hospital, Shahid Beheshti University of Medical Sciences, located in Tehran, Iran. The comprehensive clinical profiles of this patient cohort were obtained from the CRC outcomes unit database, managed by the Department of Cancer Prevention at RIGLD. The tumor samples obtained from selected patients were subjected to analysis for various parameters, including tumor location, stage, and differentiation grade, following the guidelines outlined in the American Joint Committee on Cancer Tumor-Node-Metastasis (TNM) classification [81]. Patient follow-up was conducted until July 2022, with a mean follow-up duration of 5 years (median: 5.4 years; range: 0.1–5.8 years).

### RNA extraction and qPCR analysis in Iranian tissue samples

A total of 10 ml of whole peripheral blood, as well as tumoral and adjacent healthy resected tissues, was collected from each subject. To assess both IL-2RG mRNA and miRNA expression levels, total RNA was isolated from the blood buffy coat and resected tissue samples via RNeasy mini kit (Qiagen, USA) as manufacture protocol. RNA samples underwent DNase treatment to remove DNA contamination and were examined for integrity through agarose gel electrophoresis. For mRNA expression analysis, 2 μg of RNA samples were employed for cDNA synthesis with the QuantiTect Rev transcription kit, while for miRNA expression analysis, 2 μg of RNA samples underwent cDNA synthesis using the miScript II RT Kit. Subsequently, the synthesized cDNAs were assessed using the QuantiTect SYBR® Green PCR Kit with specific primers. 18S rRNA and GAPDH (for IL-2RG), along with miR-16 and miR-1228 (for target miRNAs), were utilized as endogenous controls. The relative abundance of transcripts was calculated using REST software (Qiagen, Germany) [82]. Details about the primer sequences are demonstrated in Supplementary Table S11.

### Amplification of IL-2RG 3′ UTR fragment

The PCR reaction was performed using a Taq PCR Master Mix Kit (Qiagen, USA). The full length of 3′ UTR of IL-2RG mRNA is 335bp long. The primer sequences of 3′ UTR of IL-2RG mRNA were as follows:

5′-ACATGAAGCCTGAAACCTGAACCCC-3′ (forward), and 5′-GCCGACCATCAACAGAAACTTTATTTCT-3′ (reverse).

A Kozak sequence (ATG) was inserted within the forward primer. The cDNA synthesized sample was amplified with an initial denaturation at 94 °C for 3 min followed by 35 cycles each of 94 °C for 60 s, 60 °C for 45 s, and 72 °C for 60 s with a final extension step at 72 °C for 10 min. The PCR product was verified through 1% agarose gel electrophoresis. Next, the PCR product was extracted from the agarose gel and purified using the QIAquick Gel Extraction Kit (QIAGEN, USA), according to company protocol. Notably, all the experiments were performed in triplicate, and the calculated average number was used in statistical analysis.

### Cloning

#### Construction of vectors

The pcDNA3.1/CT-GFP-TOPO (Invitrogen, USA) and the phMGFP (Promega, USA) plasmids were chosen as shuttle vectors for expression of the 3′ UTR of IL-2RG mRNA and intended miRNAs, respectively. The pcDNA3.1/CT-GFP-TOPO vector was supplied in the linearized form with single 3′ thymidine (T) overhangs. Considering the presence of GFP as a reporter gene in both vectors, the phMGFP plasmid was digested by restriction enzymes EcoRV and XbaI (Fermentas Life Sciences, Canada), and a 698 bp fragment covering the region coding for the green fluorescent protein (GFP) was excised (PCMV + T7 EEV vector).

#### PCR cloning

For amplification of IL-2RG-related miRNAs, the primers include the coding region of pri-miRNAs and their upstream and downstream sites. Also, restriction digestion sites for EcorV and XbaI (Fermentas Life Sciences, Canada) were inserted within the forward and reverse primers of miRNAs. Details about the primer sequences and the annealing conditions for PCR amplification are shown in Supplementary Table S7. The PCR products were verified via 1% agarose gel electrophoresis, then extracted and purified from the gel by the QIAquick Gel Extraction Kit.

#### Ligation and transformation

The ligation process was set up as follows: 2 μl of each PCR product was added to a mixture of 1 μl of TOPO vector and 2 μl of salt solution (200 mM NaCl and 10 mM MgCl_2_). These reaction tubes were incubated at 22 °C for 5 min and then chilled on ice. One shot TOP10 chemically competent E. coli cells were used as the bacterial host for plasmid amplifications. 2 μl of the production from the ligation step was added to competent cells and incubated for 30 min on ice. Next, tubes were warmed for 30 s at 42 °C and chilled immediately on ice. 250 μl of room temperature liquid medium (without antibiotic) was added to the transformed cells, and tubes were shaken at 37 °C for 1 h. Then, 100 μl of these cells were spread on pre-warmed selective agar plates (with ampicillin) and incubated overnight at 37 °C.

#### Plasmids amplification and analysis

Ten colonies from each plate were picked and incubated with shaking at 37 °C in 5 ml LB medium containing 50 μg/ml ampicillin overnight. The plasmids were extracted the next day by PureLink HiPure Miniprep Kit (Invitrogen, USA). The sub-cloned fragments were subjected to automated sequencing for insertion and orientation accuracy. The primer pairs for fragment amplifications from the pcDNA3.1/CT-GFP-TOPO vector were: 5′-TAATACGACTCACTATAGGG-3′ (sense) and 5′-GGGTAAGCTTTCCGTATGTAGC-3′ (antisense).

For the phMGFP vector, the forward primer was 5′-AAGGCTAGAGTACTTAATACGA-3′ (T7 EEV promoter primer), and the specific reverse sequence for each miRNA was the same as those mentioned in Supplementary Table S7.

### Validating mRNA-miRNA interaction in recombinant HEK-293T cells with overexpressed IL-2RG 3′ UTR

#### Cell culture

The human embryonic kidney cell line 293T (HEK-293T) (ATCC CRL-11268), was chosen as the eukaryotic host. Cells were maintained and grown in Dulbecco’s Modified Eagle’s Medium (Life Technologies, CA) supplemented with 10% fetal bovine serum (FBS), at 37 °C under 5% CO_2_.

#### Transfection

HEK-293T cells were seeded in 96 well plates at the density of 1.5 × 104 cells and grown in DMEM to 80% confluency. The plasmids were diluted to 0.2 μg/100 μl of Opti-MEM I reduced-serum medium per well and mixed with 0.6 μl of FuGENE HD Transfection Reagent (Promega, USA). The mixtures were kept for 15 min at room temperature and carefully added to the cells’ medium. Transfection efficiency was evaluated 24 h later. The miRNA mimics, as well as negative control, were added to the transfected cells 72 h after GFP expression. Each well received 0.2 μg of miRNAs mimics or control diluted in 100 μl sterile water. The GFP level was monitored over the next 24 h.

#### RNA isolation and qPCR analysis in HEK-293T transfected cells

Total RNA was extracted from the HEK-293T cells (IL-2RG-transfected and empty vector-transfected), 48 h after miRNAs transfection using RNeasy Mini Kit (Qiagen, USA). The integrity of the samples was examined by agarose gel electrophoresis. The first strand cDNAs were generated with QuantiTect Rev Transcription kit according to the manufacturer’s protocol. qPCR was performed for the 3′ UTR of IL-2RG mRNA and intended miRNA using QuantiTect SYBR Green PCR Kit (Qiagen, USA). cDNA samples were amplified by 7500 Real-Time PCR System (Applied Biosystems, USA) with an initial denaturation at 95 °C for 15 min followed by 35 cycles each at 94 °C for 15 s, 54 °C for 30 s, and 72 °C for 30 s. Relative expression abundances of targets were determined by normalizing to Ribosomal 18S RNA using the 2^−∆∆CT^ method. The primer sequences used for reverse transcription and qPCR of mRNA and miRNA samples are demonstrated in Supplementary Table S6.

#### Gene drug interaction

This study utilized the Drug Gene Interaction database (DGIdb, http://www.dgidb.org/) to identify the drugs that interact with the IL-2RG gene. The DGIdb database is an online resource that compiles drug–gene interactions from various sources, including databases and text-mining [83].

### Statistical analysis

All data is represented as the mean ± S.D. (Standard deviation). A one-way analysis made comparisons between groups of variances (ANOVA) followed by an appropriate posthoc test to analyze the difference. The Mann–Whitney U test was used to assess the differences in RNA expressions between CRC patients and controls. Kaplan–Meier curves were constructed to demonstrate the overall survival and compared using the log-rank test. Cox’s proportional hazards model was also used for univariate and multivariate logistic regression. Spearman test was employed to analyze the relationship between the differential expression of the target RNAs and other variables. All statistical analyses were made using the IBM SPSS Statistics software version 22 (IBM, USA). A p-value less than 0.05 was considered statistically significant.

### Supplementary Information

Supplementary Material 1.

Supplementary Material 2.

## Data Availability

The datasets used in this study can be found in online repositories, the name of which can be found in the article. Other data presented in this study are available on request from the corresponding authors.
