# Supplementary material for: IL-2RG as a possible immunotherapeutic target in CRC predicting poor prognosis and regulated by miR-7-5p and miR-26b-5p
Source: J Transl Med. 2024 May 8;22:439. doi: 10.1186/s12967-024-05251-2 (PMC11080123; doi:10.1186/s12967-024-05251-2)
Supplement: Supplementary file 1 — Supplementary Material 1. [file 12967_2024_5251_MOESM1_ESM.docx]

**Supplementary Figure 1| Expression Analysis of the IL-2RG gene in CRC tissue.** Dot plot of the IL-2RG transcript expression in pan-cancer compared to normal tissues.

**Supplementary Figure 2| Protein expression of IL-2RG in tumor cells compared to normal enterocyte cells.**

**Supplementary Figure 3| Association between IL-2RG transcript expression and IL-2RG promotor methylation based on 522 colorectal Adenocarcinoma (TCGA, PanCancer Atlas) patients retrieved from the CbioPortal database.** **P* < 0.05, ***P* < 0.01, ****P* < 0.001. *P* < 0.05 was considered significant.

**Supplementary Figure 4| Co-expression genes analysis.** Volcano Plot of positively and negatively correlated genes of IL2RG.


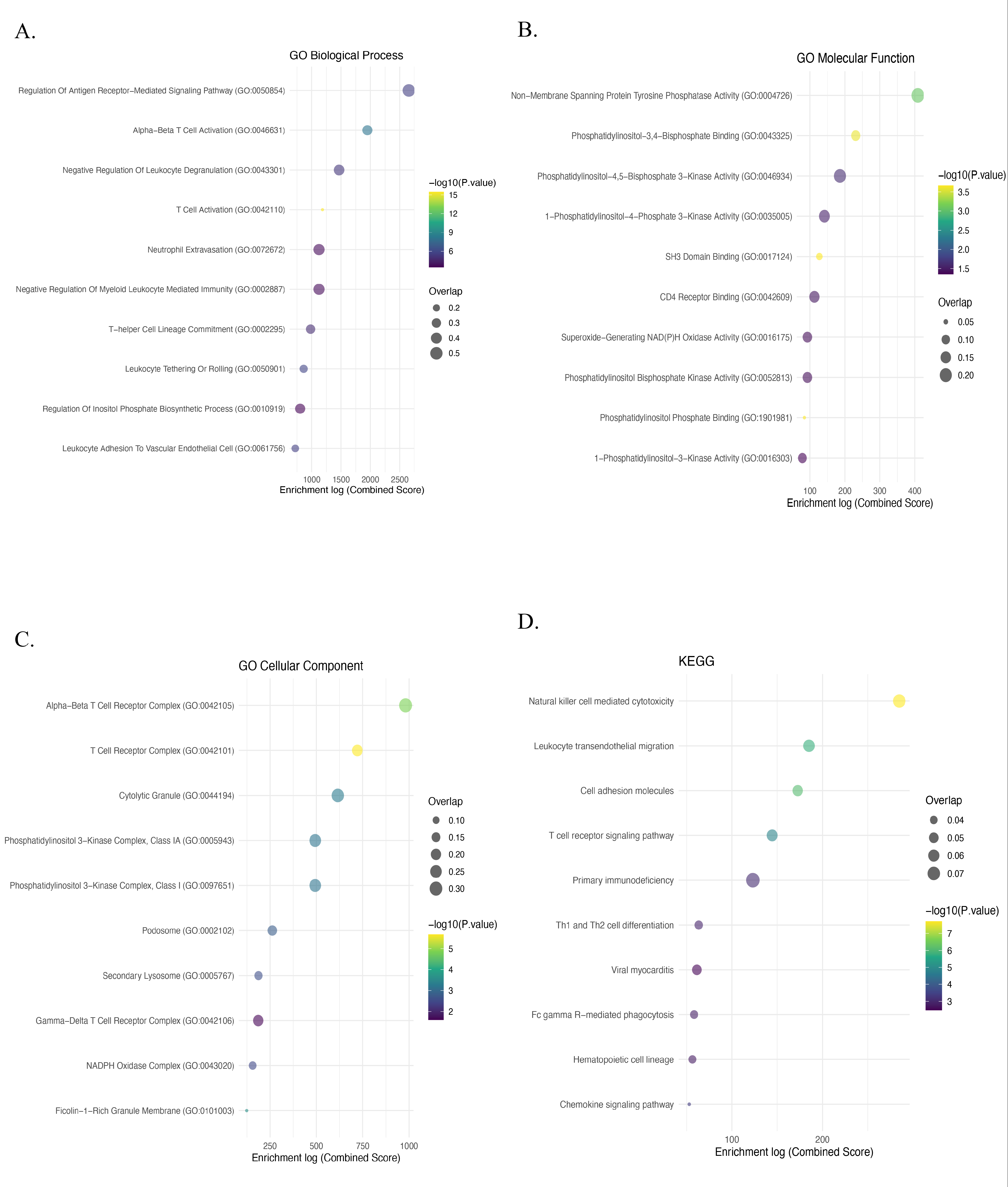


**Supplementary Figure 5|** Gene Ontology analysis of IL-2RG: Biological Process **(A)**, Molecular Function **(B)**, and Cellular Component **(C)**. **(D)** KEGG pathway analysis.

**Supplementary Figure 6| Cloning of the 3′ UTR region of human IL2RG mRNA into the pcDNA3.1/CT-GFP-TOPO vector.** (a) The amplified segment of IL2RG mRNA including the 3′ UTR region of human IL2RG mRNA (Black) along with the Kozak sequence; (b) Construction of the pcDNA3.1/CT-GFP-TOPO vector before and after insertion (pcDNA3.1/CT-IL2RG).

.

**Supplementary Figure 7| Complementary sequences of intended miRNAs with the 3′ UTR region of human IL2RG mRNA.** (a) let-7a; (b) miR-7; (c) miR-26b; (d) miR-128; (e) miR-421 and (f) miR-873

**Supplementary Figure 8| Transfection of pcDNA3.1/CT-IL2RG into the HEK-293T cells.** (a) Non-transfected (NT); (b) Transfected cells with the pcDNA3.1/CT-GFP-TOPO vector and (c) Transfected cells with the pcDNA3.1/CT-IL2RG. (d) The qPCR analysis of the human hγC and target miRNAs. Scale bar: 50 μm. Each value of qPCR results is the mean±S.D. of three separate experiments.

**Supplementary Figure 9|** The pan-cancer expression analysis of the lncRNAs (SNHG16, MEG3, XIST, SOX21-AST, ZFAS7, SNHG11, OIP5-AST1, SNHG5, SPINT1-AS1, SNHG1, NEAT1, HCG11, NORAD, SNHG14, LINC00174, DLX6-AS1, SNHG6, MALAT1, TUG1, LINC00665, and KCNQ10T1) based on TCGA data.


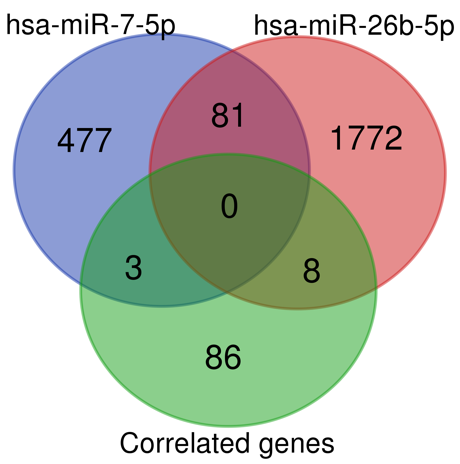


**Supplementary Figure 10| Putative targets of miRNA-7 and miRNA-26b among 100 coexpressed genes.** The intersection between mRNAs targeted by hsa-miR-7-5p (blue circle) and targeted by hsa-miR-26b-5p (red circle) with 100 co-expressed genes of IL2rg via Venn diagram.
